# Laparoscopic exploration in pediatric surgery emergencies

**Published:** 2010-02-25

**Authors:** I Drăghici, L Drăghici, M Popescu, M Liţescu

**Affiliations:** * Department of Pediatric Surgery, ‘Maria Sklodowska Curie’ Hospital, BucharestRomania; ** Department of General Surgery, ‘Sf. Ioan’ Hospital, National Center of Laparoscopic Training, BucharestRomania

**Keywords:** surgical pediatric emergencies, exploratory laparoscopy, laparoscopic approach

## Abstract

The laparoscopic approach of pediatric surgery emergencies represents a specific preoccupation in hospitals everywhere in the world. Nowadays, when confronted with this pathology, pediatric surgeons are able to apply certain well–defined therapeutic protocols, depending on the technical equipment at their disposal and their laparoscopic expertise and training.

We hereby present some of the surgical pediatric emergencies that have been subjected to minimally invasive celioscopic techniques, in the Department of Pediatric Surgery ‘Maria Sklodowska Curie’ Hospital, from August 1999 to July 2007. Out of 83 exploratory laparoscopies, 12 were performed for emergency pathology, other than acute appendicitis (in its various forms, including peritonitis) or acute cholecystitis. However, during the above–mentioned period, the number of therapeutic laparoscopies for emergencies has grown significantly (239 from a total of 663 laparoscopies), reflecting to a large extent the activity of a clinic with an emergency surgery profile.

The authors conclude that exploratory laparoscopies in pediatric surgery emergencies are suited for surgical teams with a solid experience in celioscopy and a certain professional maturity, necessary to correctly appreciate the surgical and anesthetic risks involved by each individual case.

It is not recommended that inexperienced laparoscopic surgeons embark on the ‘adventure’ of this minimally invasive approach for this type of pathology. Only when the training and learning process is fully and correctly completed, specialists are offered the advantage of continuing a celioscopic exploration by performing a minimally invasive therapeutic procedure, even for a pediatric emergency case.

## Introduction

There are undeniable advantages in this minimally invasive approach to the pediatric surgical emergency pathology. Laparoscopy reduces the number of general complications that can occur in rare situations during surgery. Because of these incidents, surgeons are highly recommended to be prudent when they are required to appreciate the surgical and anesthetic risks for each individual case. When choosing to perform a surgical treatment with the help of laparoscopy, the doctor must take into consideration the risks and benefits of the procedure for the patient [1].

Although appendicitis represents the most frequent pediatric surgical emergency, laparoscopic procedures have also been performed successfully in other pathologies such as: genital, intestinal obstruction, acute pancreatitis and acute posttraumatic abdomen. Its utility is undisputed, being most commonly indicated to obese children, to those with chronic abdominal pain that becomes acute and especially to girls at puberty for whom there is a suspicion of ovarian pathology [2– 5].

## Material and methods

The laparoscopic activity of our department quickly adapted to the requirements imposed by the status of an emergency hospital, embracing the tendency towards minimally invasive explorations in different types of acute pathology, in a short period of time.

In the Department of Surgery ‘Maria Sklodowska Curie’ Hospital, we registered a number of 12 exploratory laparoscopies for emergency pathology other than acute appendicitis (in its different forms, including peritonitis) or acute cholecystitis, from August 1999 to July 2007. The number of therapeutic laparoscopies in emergency cases has nevertheless been much higher (239 from a total of 663 laparoscopies). The cases in which the positive diagnostic was corroborated with the help of surgical records, clinical examination and radiological exams (intestinal obstructions due to scar abdomens, peritonitis and genital pathology diagnosed with the help of ultrasound and clinic, complications occurred in intra abdominal tumors–hemorrhage, sepsis) have been excluded from the present study. In all these cases, laparoscopy was performed as a minimally invasive therapeutic radical or palliative procedure.

The exploratory laparoscopy has been deemed necessary in 12 situations in which the clinical findings, radiological and laboratory studies were not accurate. The surgical method applied (open surgery or minimally invasive) to these cases does not make the object of our study, but we are now able to confirm that the laparoscopic treatment of this kind of pathology is useful and is applied in our clinic, having very good results.

## Results

Table 1 contains different groups of pediatric surgical emergencies that were diagnosed through an exploratory laparoscopy.

**Table 1:** Pediatric surgical emergencies explored laparoscopically

Surgical emergencies	Intraoperative diagnosis	Number of cases	Number of cases / pathology	% of total exploratory laparoscopy (83)
Genital pathology	Ovarian breached cyst (hemorrhage)	3	5	6,00%
	Fallopian tube and ovarian torsion	1		
	Pelvic peritonitis	1		
Intestinal obstruction	Intestinal volvulus	1	4	4,80%
	Adherent bands	3		
Acute pancreatitis	Acute edematous pancreatitis	2	2	2,40%
Acute posttraumatic abdomen	Hepatic lesion (intraperitoneum hemorrhage)	1	1	1,20%

Regarding emergencies related to the genital area, we have been confronted with three cases of intra peritoneum hemorrhage due to rupture of ovarian cysts. Only one of these incidents was significant, i.e. a 13–year–old female patient (hemodynamically stable) whose abdominal ultrasound was irrelevant, but the suggestive clinical findings for intra peritoneum hemorrhage and acute surgical abdomen imposed an exploratory laparoscopy. The case has been resolved in a minimally invasive manner.

The cases of intestinal obstruction which were difficult to diagnose before surgery, proved to be mainly abdominal pains with no signs of peritoneum irritation or meteorism. We can therefore affirm that the intraoperative diagnosis came as a surprise. Consequently, the intestinal volvulus as a direct cause of lymphatic mesenteric cyst needed surgical conversion to open surgery, while the adherence syndromes occurring after classical appendectomies were treated laparoscopically.

**Diagram 1:**
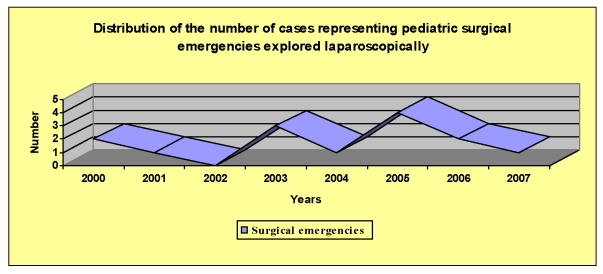
Case progression

Although the laboratory diagnosis of acute pancreatitis is relatively easy to establish, its etiology is sometimes controversial. In the case of one acute pancreatitis that had a slow evolution despite the long–term medical treatment, we decided to perform an exploratory laparoscopy. The biochemical exams have been completed by the analysis of the peritoneum fluid and ganglion–peritoneum biopsy followed by immunological exams. After three weeks from the admission date, the virus etiology of the pancreatitis has been established. The case progressed slowly towards healing.[Diagram 1]

The thoracic abdominal traumatology treated in our clinic is vast. Most of the posttraumatic acute abdomens need specific surgical treatment and are usually explored by open surgery. This classical approach is justified by the large amount of lesions and hemodynamic instability, which is a contraindication in laparoscopy. In the case of an 11–year–old female patient, who has suffered a stab wound in the right hypochondrium, the surgical team opted for an exploratory laparoscopy, which identified a superficial lesion of the hepatic parenchyma. The surgical solution applied was haemostasis with electro coagulation and peritoneal drain.

## Discussions

Employing exploratory laparoscopies in pediatric surgery emergencies is suited for surgical teams with a solid experience in celioscopy and a certain professional maturity, necessary to correctly appreciate the surgical and anesthetic risks involved by each individual case. 

It is not recommended that inexperienced laparoscopic surgeons embark on the ‘adventure’ of this minimally invasive approach for this type of pathology. Only when the training and learning process is fully and correctly completed, specialists are offered the advantage of continuing a celioscopic exploration by performing a minimally invasive therapeutic procedure, even for a pediatric emergency case.

For the cases of acute abdomen when imagistic examinations are not conclusive, we recommend the celioscopic exploration. With this procedure, we can easily establish the differential diagnosis of peritonitis and of other acute female genital area conditions (ectopic pregnancy, ovarian breached cyst or torsion).

The recent progress of ultrasound exploration and the dosage of blood beta HCG allow an early diagnosis of ectopic pregnancy. On the other hand, certitude in diagnosis is only given by the exploratory laparoscopy [6]. The rare cases of ectopic ruptured pregnancy in our department, have been treated classically (through open surgery) due to the hemodynamic instability of young patients who did not permit a safe laparoscopy. Nevertheless, the minimally invasive method is in principle recommended, and an early diagnosis would lead to a conservative laparoscopic treatment capable to maintain the fertility of the patient. 

An intra peritoneum hemorrhage occurred, following a ruptured ovarian cyst is a typical example of a patient explored in emergency for symptoms suggesting acute genital pathology. The clinical examination, previous relevant ultrasounds and a stable blood pressure create the premises for a minimally invasive approach even if the duration of the surgery is prolonged by the cleansing and aspiration of the peritoneal cavity. At the end of the laparoscopic exploratory intervention, the surgeon must be able to exclude any sign that might suggest malignity of the ovarian cyst (intracystic vegetations) and by exploring the uterus and ovary on the other side to ensure that a conservatory treatment (celioscopic or open surgery) can be initiated.

**Fig 1:**
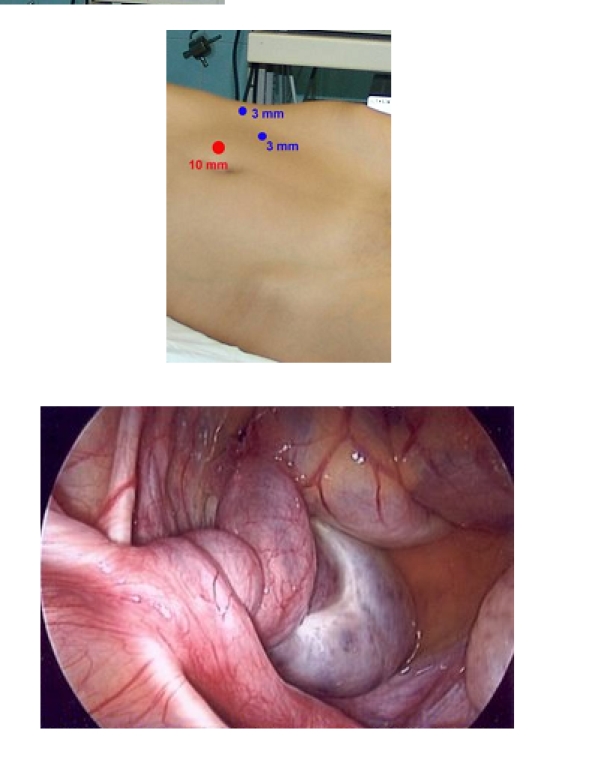
Fallopian torsion: laparoscopic approach

The fallopian tube and ovary torsion should be suspected in patients with acute pain, recurrent in time, localized in one of the two inferior quadrants frequently irradiating towards the external genital organs [7]. Doppler color ultrasound represents an excellent diagnostic method [8]. Intraoperatively, it is mandatory to examine the ovary and fallopian tube on the opposite side. According to Davis and Feins [9], as well as to Grunewald [10], the contralateral oophoropexy must be taken into account in the rare cases of asyncronic torsion of the normal ovary. The torsion is frequently associated with tumors (cystic and solid) and must be investigated accordingly. The treatment consists in rotating the fallopian tube back and checking its viability to be sure of the gonad preservation Fig 1

**Fig 2:**
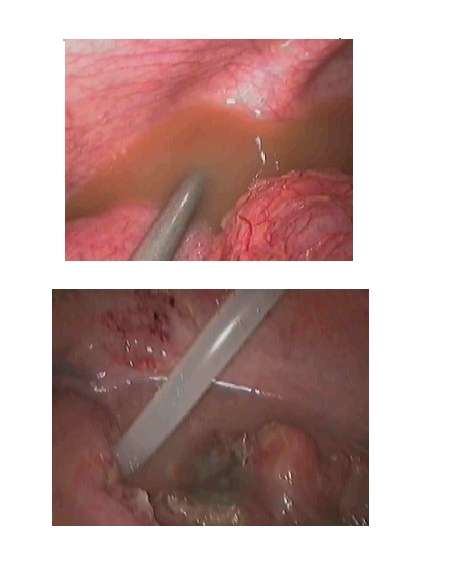
Laparoscopic treatment of pelvic peritonitis

Laparoscopy also occupies a crucial place in the diagnosis of upper genital infections. In the early stages of infection, the clinical and biological diagnosis has a high rate of false negative and false positive results. Exploratory celioscopy permits the complete evaluation of the pelvis and represents the first stage of the treatment (Fig 2). Laparoscopy is always preceded by the extraction of the peritoneal fluid for the bacteriological exam. Pelvic examination becomes crucial for a case of acute abdomen of suspected genital etiology. The uterus is pushed to the front with the help of the left hand clamp and the Douglas is the first to be explored. Afterwards, each fallopian tube and ovary is mobilized and gently inspected using blunt clamps. This instrumental inspection represents an important step in the diagnosis of early stage salpingitis described by a rigid fallopian tube. If a uterine tube is slightly distended, suggestive for a salpinx beginning to suppurate, biopsy is the standard procedure applied. Sometimes, the simple compression of the tube on the pelvic wall externalizes the pus [11].

### Clinical case Ⅰ

A four–year–old patient is admitted for diffuse abdominal pain in the upper quadrant, associated with non–alimentary vomiting and moderate meteorism. Abdominal X ray reveals hydro aerated images of the small bowl. The ultrasound confirms a cystic tumor of small dimensions located in the center of the abdomen. The decision taken is to perform an exploratory laparoscopy that diagnoses intestinal obstruction through volvulus of a lymphatic mesenteric cyst. The volvulus is caught in an important adherent bloc and the tumor cannot be celioscopically untwisted, therefore the surgeon makes the correct decision to resort to open surgery.

What is the reason for which the surgeon chose this additional method of diagnosis of the obstructive cystic tumor, already detected before the surgery? Most certainly, the disproportion between the dimensions of the tumor and the severity of the symptoms, determined our colleague to treat with extra care an apparently ‘simple cyst’. Only the celioscopic image established the real and surprising explanation for the intense and complex symptoms, the volvulus.

As a conclusion, the surgeons’ preoccupation for the minimally invasive approach in pediatric emergencies also refers to the suspicions of intestinal obstruction, in which the hydro aerated images are minimal to medium.

**Fig 3:**
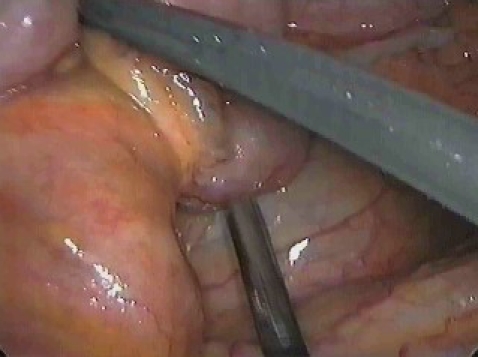
Laparoscopic untwisting of the volvulus

**Fig 4:**
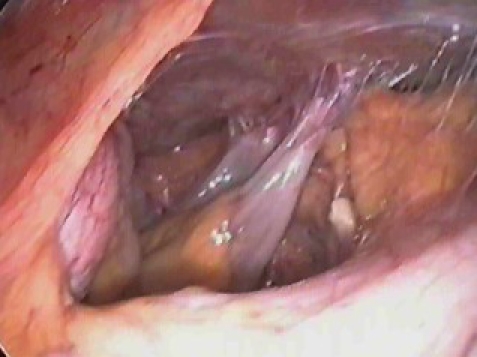
Intestinal adherences with bowl obstruction

The most frequent cause of obstruction of a pre-operated abdomen is the intra-peritoneal adherences (Fig 3,Fig 4). An ‘old’ obstruction however, due to the intense distension of the abdomen that it generates, represents an obstacle to the laparoscopic method, because of the lack of operating space and the high risk of intestine lesions. In our study, we encountered only one laparoscopic exploration for a case of occlusive syndrome installed late after an appendectomy. The case was treated entirely laparoscopically. However, we came across many postoperative obstructive adherences treated celioscopically as a therapeutic method for sectioning the ‘straps’ and peritoneal drain. The latter have not been included in the study because the surgeon did not have any doubts regarding the pre–operatory clinical and radiological diagnosis.

### Clinical case Ⅱ

An eleven–year–old patient is admitted in the emergency room for a right hypocondrium stab wound, accidentally inflicted with a sharp object. The surgeon cannot establish with certainty if the lesion penetrated or perforated any abdominal organs, hence he decides to perform an exploratory laparoscopy. The minimally invasive procedure detects a superficial lesion of the hepatic parenchyma on the diaphragmatic face of the right lobe. No other organs were injured. The surgical solution, also minimally invasive, consisted in haemostatic mono polar electric coagulation and peritoneum cavity drain for 24 hours. The postoperative evolution was favorable and no complications occurred.

The surgeon chose this type of exploration first in an attempt to avoid a traumatizing and unaesthetic large classical incision in a pediatric female patient, who also benefited from the advantage of a good hemodynamic equilibrium.

**Fig 5:**
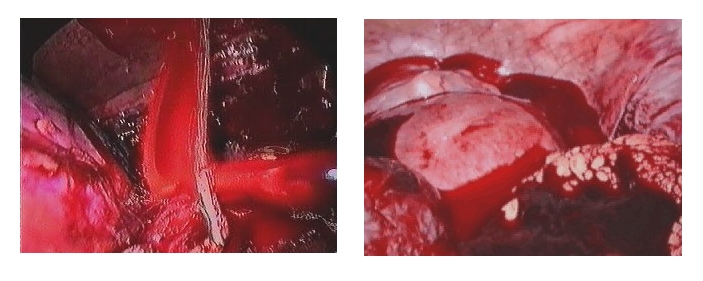
Intra peritoneum hemorrhage in lesion of renal pedicle – exploratory laparoscopy

Due to the emergency status of our hospital, surgeons tend to prefer the invasive approach. Yet, once the celioscopic techniques have been introduced, the manner of addressing thoracic and abdominal trauma has been revised. The presented case is a good example of a medium amplitude trauma, which we recommend to be laparoscopically explored. On the contrary, it is not recommended to treat patients with severe physical trauma (road accident casualties, sport injuries, ‘white weapons’ aggressions) through minimally invasive techniques (Fig 5). The large incision of an open surgery has both an exploratory and a therapeutic purpose, allowing a good surgical haemostasis of important vessels pedicle and organs.

Phillip Rossi and David Mullins from Dallas, Texas, published a prospective research, carried out on 32 abdominal penetrating and non–penetrating traumas, subjected to exploratory celiotomy. After using celioscopy as a first procedure in all these cases, a 16%–19% error has been noticed in identifying lesions that have been later detected by classic surgery. There were multiple areas with lesions, which remained undetected laparoscopically: liver, pancreas, stomach, duodenum small bowl, mesentery, ureter and urinary bladder. Subsequent complications emerged in 6.25% of cases [12]. 

It is certain that there are different abdominal areas that cannot be explored in detail through laparoscopy, but the celioscopic evaluation of penetrating abdominal trauma has nevertheless a high rate of accuracy. 

Even though diagnostic laparoscopy has a reduced sensitivity (<50%) for superficial lesions of intra–abdominal organs, it has an excellent sensitivity (96.2%) and specificity (100%) to establish the need of a therapeutic open surgery.

## Conclusions

- Exploratory laparoscopy represents a good diagnostic method of surgical pediatric emergencies.
- The celioscopic management of emergencies is suited for surgeons with a solid experience in exploratory laparoscopy.
- The hemodynamic shock represents an absolute contraindication for the method, constituting the main selection criterion for laparoscopic exploration.
- Laparoscopy took over an important part of the open surgery indications.
- Acute genital pathology is frequently subjected to this method, with good results and considerable benefits.
- Peritonitis and neglected old occlusions do not draw a benefit from the application of the celioscopic method.
- The acute posttraumatic abdomen is ‘a slippery slope’ for the laparoscopic method. There is a risk of omitting lesions of different organs, especially those of the small bowl, colon and retro–peritoneum area.
